# Fatigue data for polyether ether ketone (PEEK) under fully-reversed cyclic loading

**DOI:** 10.1016/j.dib.2016.01.052

**Published:** 2016-02-03

**Authors:** Rakish Shrestha, Jutima Simsiriwong, Nima Shamsaei

**Affiliations:** a Department of Mechanical Engineering, Mississippi State University, Box 9552, Mississippi State, MS 39762, USA; b Center for Advanced Vehicular Systems (CAVS), Mississippi State University, Box 5405, Mississippi State, MS 39762, USA

**Keywords:** Fatigue, Cyclic deformation, Thermoplastic, Polyether ether ketone, Strain-life experiments, Frequency effects

## Abstract

In this article, the data obtained from the uniaxial fully-reversed fatigue experiments conducted on polyether ether ketone (PEEK), a semi-crystalline thermoplastic, are presented. The tests were performed in either strain-controlled or load-controlled mode under various levels of loading. The data are categorized into four subsets according to the type of tests, including (1) strain-controlled fatigue tests with adjusted frequency to obtain the nominal temperature rise of the specimen surface, (2) strain-controlled fatigue tests with various frequencies, (3) load-controlled fatigue tests without step loadings, and (4) load-controlled fatigue tests with step loadings. Accompanied data for each test include the fatigue life, the maximum (peak) and minimum (valley) stress–strain responses for each cycle, and the hysteresis stress–strain responses for each collected cycle in a logarithmic increment. A brief description of the experimental method is also given.

## Specifications Table

**Table:** 

Subject area	Engineering
More specific subject area	Fatigue of polymers
Type of data	Table (Microsoft Excel file format)
How data was acquired	Strain-controlled and load-controlled fatigue experiments (laboratory)
Data format	Raw and analyzed
Experimental factors	Cylindrical dog-bone specimens were first machined and polished in different stages to remove any machining marks.
Experimental features	Uniaxial fully-reversed fatigue tests under strain-controlled or load-controlled mode
Data source location	Center for Advanced Vehicular Systems (CAVS), Mississippi State University, Box 5405, Mississippi State, MS 39762, USA
Data accessibility	Data is within this article.

## Value of the data

• The data provided in this paper are the results of the experimental investigation using the *ε*–*N* approach to obtain the fatigue properties of PEEK thermoplastic, which can be used to validate various fatigue models for polymers.
• The presented data provide the overall cyclic deformation and fatigue behavior of PEEK polymer under different cyclic loading modes. The test method can be generalized for other semi-crystalline polymers.
• The stress–strain responses provided in this paper can be used to obtain the frequency effects on PEEK fatigue behavior.

## 1 Data

The presented data sets are categorized into four Microsoft Excel workbooks according to the type of tests. The workbook named (1) “Nominal Temperature,” contains the strain-controlled fatigue tests with adjusted frequency to achieve nearly fixed strain rates, and thus, similar nominal temperature rise in all fatigue tests, (2) “Frequency Effect Tests,” contains the strain-controlled fatigue tests with various frequencies, (3) “Load-Controlled Test,” contains load-controlled fatigue tests under constant amplitude loadings, and (4) “Load-controlled Step Test,” contains load-controlled fatigue tests with step loadings. A summary of each test with corresponding strain/stress amplitudes, test frequency, specimen name, and fatigue life are presented in Table 1, Table 2 for strain-controlled and load-controlled tests, respectively. The data have been deposited to the Data in Brief Dataverse:10.7910/DVN/YSFURO.

## 2 Experimental design, materials and methods

The study was conducted on a neat PEEK polymer [1]. Fatigue specimens were machined using a CNC lathe to produce a cylindrical dog-bone shape with the gage diameter of 6.35 mm and gage length of 18 mm following ASTM E606-04 standard [2]. The specimens were further polished using different grit sand papers to remove any mark from machining on the gage section of the specimen.

All of the uniaxial fully-reversed fatigue tests were conducted under strain-controlled or load-controlled loading condition following the ASTM D7791 standard [3]. The fatigue tests were performed using MTS 858 closed-loop servo hydraulic load frame with a 25 kN load cell. The strain introduced on the gage section of the specimen was obtained using a MTS axial extensometer with a gage length of 15 mm. Due to high damping characteristic of polymers, the rise in temperature in PEEK specimens is sensitive to the test frequency (i.e. strain rate) and strain/load amplitude. Thus, a laser thermometer was used to monitor the temperature on the gage section of the specimen during fatigue tests [1].

## Figures and Tables

**Table 1:** Summary for uniaxial fully-reversed (*R*=−1) strain-controlled fatigue tests.

**Specimen ID**	**Strain amplitude,**$\varepsilon_{a}$**(mm/mm)**	**Frequency (Hz)**	**Reversals to failure, 2*****N***_***f***_
*Nominal Temperature Tests*			
S50	0.02	3	1,449,114
S21	948,248
S22	946,032
S19	0.025	1	475,810
S30	231,964
S31	179,018
S46	0.03	0.75	208,896
S48	125,856
S47	124,030
S42	0.035	0.5	92,078
S43	48,090
S35	46,772
S24	0.04	0.5	18,454
S25	14,172
S23	7766

*Frequency Effect Tests*			
S20	0.02	1	1,723,898
S94	1,460,066
S55	0.025	2	437,176
S26	0.03	0.5	51,472
S28	15,716
S53	1	216,650
S59	0.035	0.25	32,076
S34	0.75	53,968
S4	1	155,206
S8	86,514
S56	0.04	0.25	4924
S95	4306

**Table 2:** Summary for uniaxial fully-reversed (*R*=−1) load-controlled fatigue tests.

**Specimen ID**	**Stress amplitude, *σ_a_* (MPa)**	**Frequency (Hz)**	**Reversals to failure, 2*N_f_***

*Load-Controlled Tests*			
S61	45	0.75	>2,000,000
S71	70	1	95,830^a^
S72	70	2	109,294^a^
S68	80	0.5	6444^a^
S69	80	0.75	7404^a^
S70	80	1.5	9716^a^

*Load-Controlled Step Tests*			
S65	100–45	0.4	172^a^
S64	100–45	0.4	306^a^
S66	100–45	0.6	>52,810
S63	100–45	0.75	>2,000,000

a Specimen failed due to necking.
