# Supplementary material for: Fatigue data for polyether ether ketone (PEEK) under fully-reversed cyclic loading
Source: Data Brief. 2016 Feb 3;6:881–4. doi: 10.1016/j.dib.2016.01.052 (PMC4752734; doi:10.1016/j.dib.2016.01.052)
Supplement: Supplementary file 1 — Supplementary material [file mmc1.pdf]

The present data in this article includes:

- The workbook named “Nominal Temperature,” which contains the data obtained from the uniaxial fully-reversed strain-controlled fatigue tests in which the rise in temperature at the gage section of the specimen was maintained constant for all tests, by adjusting frequency (i.e. strain rate).
- The workbook named “Frequency Effect Tests,” which includes the data obtained from the uniaxial fully-reversed strain-controlled fatigue experiments conducted at selected strain amplitudes and at various frequencies to investigate the effects of frequency (i.e. strain rate) on the fatigue behavior of PEEK thermoplastic.
- The workbook named “Load-Controlled Test,” which contains the data obtained from the uniaxial fully-reversed load-controlled fatigue tests to compare the PEEK fatigue behavior under load-controlled and strain-controlled modes.
- The workbook named “Load-controlled Step Test,” which contains the data obtained from the uniaxial fully-reversed load-controlled tests with step loadings to duplicate the stress responses due to the cyclic softening, which was observed in the strain-controlled fatigue tests.

It is to be noted that the specimen name presented in this article and data files are consistent with the name presented in [1]. Additionally, two worksheets are included in this article for each specimen. The first worksheet is denoted by the “Cyclic Deformation,” which contains time, segments (reversals), and hysteresis stress-strain responses, which are recorded for each collected cycle in a logarithmic increment. Some of the tests included in these worksheets were interrupted due to sudden drop in the applied load that triggered the test machine to stop. These tests were resumed and continued until the fatigue failure of the specimens. The second worksheet is denoted

by “PeakValley,” which contains the maximum (peak) and minimum (valley) stress-strain responses for all cycles, as well as the corresponding time and segments (reversals).
